# Watching the brain in action

**DOI:** 10.7554/eLife.00866

**Published:** 2013-05-28

**Authors:** Bradford Z Mahon

**Affiliations:** 1 **Bradford Z Mahon** is at the Department of Brain and Cognitive Sciences, and the Department of Neurosurgery, University of Rochester, Rochester, United Statesmahon@rcbi.rochester.edu

**Keywords:** fMRI, tool use, intentions, action, neuroscience, motor, Human

## Abstract

Functional magnetic resonance imaging has been used to identify the different networks in the brain that underpin the use of tools by humans.

**Related research article** Gallivan JP, McLean DA, Valyear KF, Culham JC. 2013. Decoding the neural mechanisms of human tool use. *eLife* **2**:e00425. doi: 10.7554/eLife.00425

**Image** Certain brain regions respond preferentially to viewing tools and planning tool actions (red) or to viewing the human body and planning hand actions (green)

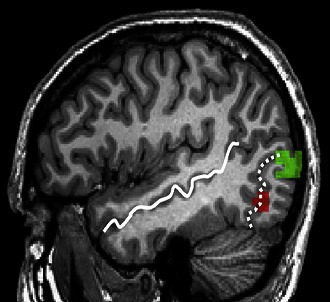

In our daily lives, we interact with a vast array of objects—from pens and cups to hammers and cars. Whenever we recognize and use an object, our brain automatically accesses a wealth of background knowledge about the object’s structure, properties and functions, and about the movements associated with its use. We are also constantly observing our own actions as we engage with objects, as well as those of others. A key question is: how are these distinct types of information, which are distributed across different regions of the brain, integrated in the service of everyday behavior? Addressing this question involves specifying the internal organizational structure of the representations of each type of information, as well as the way in which information is exchanged or combined across different regions. Now, in *eLife*, Jody Culham at the University of Western Ontario (UWO) and co-workers report a significant advance in our understanding of these ‘big picture’ issues by showing how a specific type of information about object-directed actions is coded across the brain (Gallivan et al., 2013b).

A great deal is known about which brain regions represent and process different types of knowledge about objects and actions (Martin, 2007). For instance, visual information about the structure and form of objects, and of body parts, is represented in ventral and lateral temporal occipital regions (Goodale and Milner, 1992). Visuomotor processing in support of object-directed action, such as reaching and grasping, is represented in dorsal occipital and posterior parietal regions (Culham et al., 2003). Knowledge about how to manipulate objects according to their function is represented in inferior-lateral parietal cortex, and in premotor regions of the frontal lobe (Culham et al., 2003; Johnson-Frey, 2004).

**Figure 1.**
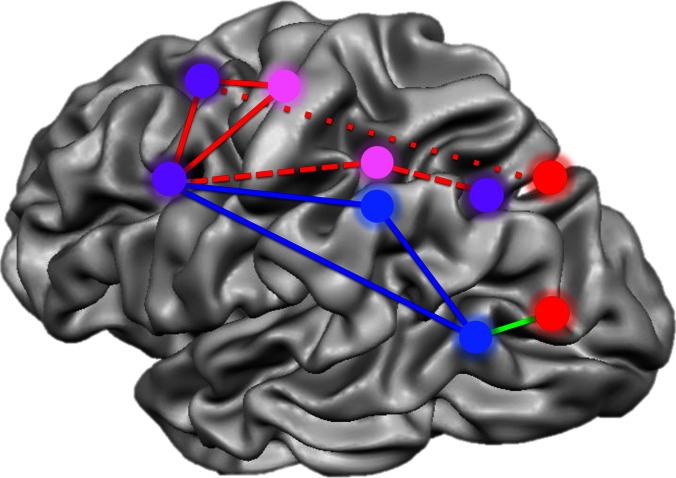
Summary of the networks of brain regions that code for movements of hands and tools. By comparing brain activation as subjects prepared to reach towards or grasp an object using their hands or a tool, Gallivan et al. identified four networks that code for distinct components of object-directed actions. Some brain regions code for planned actions that involve the hands but not tools (red), and others for actions that involve tools but not the hands (blue). A third set of regions codes for actions involving either the hands or tools, but uses different neural representations for each (pink). A final set of areas code the type of action to be performed, distinguishing between reaching towards an object as opposed to grasping it, irrespective of whether a tool or the hands alone are used (purple). The red lines represent the frontoparietal network implicated in hand actions, with the short dashes showing the subnetwork involved in reaching, and the long dashes, the subnetwork involved in grasping. The blue solid lines show the network implicated in tool use, while the green line connects areas comprising a subset of the perception network.

FIGURE CREDIT: IMAGE ADAPTED FROM FIGURE 7 IN GALLIVAN ET AL., 2013B

Culham and colleagues—who are based at the UWO, Queen's University and the University of Missouri, and include Jason Gallivan as first author—focus their investigation on the neural substrates that underlie our ability to grasp objects. They used functional magnetic resonance imaging (fMRI) to scan the brains of subjects performing a task in which they had to alternate between using their hands or a set of pliers to reach towards or grasp an object. Ingeniously, the pliers were reverse pliers—constructed so that the business end opens when you close your fingers, and closes when your fingers open. This made it possible to dissociate the goal of each action (e.g., ‘grasp’) from the movements involved in its execution (since in the case of the pliers, ‘grasping’ is accomplished by opening the hand).

Gallivan et al. used multivariate analyses to test whether the pattern of responses elicited across a set of voxels (or points in the brain) when the participant reaches to touch an object can be distinguished from the pattern elicited across the same voxels when they grasp the object. In addition, they sought to identify three classes of brain regions: those that code grasping of objects with the hand (but not the pliers), those that code grasping of objects with the pliers (but not the hand), and those that have a common code for grasping with both the hand and the pliers (that is, a code for grasping that is independent of the specific movements involved).

One thing that makes this study particularly special is that Gallivan et al. performed their analyses on the fMRI data just ‘before’ the participants made an overt movement. In other words, they examined where in the brain the ‘intention’ to move is represented. Specifically, they asked: which brain regions distinguish between intentions corresponding to different types of object-directed actions? They found that certain regions decode upcoming actions of the hand but not the pliers (superior-parietal/occipital cortex and lateral occipital cortex), whereas other regions decode upcoming actions involving the pliers but not the hand (supramarginal gyrus and left posterior middle temporal gyrus). A third set of regions uses a common code for upcoming actions of both the hands and the pliers (subregions of the intraparietal sulcus and premotor regions of the frontal lobe).

The work of Gallivan et al. significantly advances our understanding of how the brain codes upcoming actions involving the hands. Research by a number of teams is converging to suggest that such actions activate regions of lateral occipital cortex that also respond to images of hands (Astafiev et al., 2004; Peelen and Downing, 2005; Orlov et al., 2010; Bracci et al., 2012). Moreover, a previous paper from Gallivan and colleagues reported that upcoming hand actions (grasping versus reaching with the fingers) can be decoded in regions of ventral and lateral temporal-occipital cortex that were independently defined as showing differential BOLD responses for different categories of objects (e.g., objects, scenes, body parts; Gallivan et al., 2013a). Furthermore, the regions of lateral occipital cortex that respond specifically to images of hands are directly adjacent to those that respond specifically to images of tools, and also exhibit strong functional connectivity with areas of somatomotor cortex (Bracci et al., 2012).

Taken together, these latest results and the existing literature point toward a model in which the connections between visual areas and somatomotor regions help to organize high level visual areas (Mahon and Caramazza, 2011), and to integrate visual and motor information online to support object-directed action. An exciting issue raised by this study is the degree to which tools may have multiple levels of representation across different brain regions: some regions seem to represent tools as extensions of the human body (Iriki et al., 1996), while other regions represent them as discrete objects to be acted upon by the body. The work of Gallivan et al. suggests a new way of understanding how these different representations of tools are combined in the service of everyday behavior.
